# A Transductive Model-based Stress Recognition Method Using Peripheral Physiological Signals

**DOI:** 10.3390/s19020429

**Published:** 2019-01-21

**Authors:** Minjia Li, Lun Xie, Zhiliang Wang

**Affiliations:** School of Computer and Communication Engineering, University of Science and Technology Beijing, Beijing 100083, China; b20160292@xs.ustb.edu.cn (M.L.); wzl@ustb.edu.cn (Z.W.)

**Keywords:** stress recognition, peripheral physiological signals, neighborhood knowledge, transductive SVR, learning scenario

## Abstract

Existing research on stress recognition focuses on the extraction of physiological features and uses a classifier that is based on global optimization. There are still challenges relating to the differences in individual physiological signals for stress recognition, including dispersed distribution and sample imbalance. In this work, we proposed a framework for real-time stress recognition using peripheral physiological signals, which aimed to reduce the errors caused by individual differences and to improve the regressive performance of stress recognition. The proposed framework was presented as a transductive model based on transductive learning, which considered local learning as a virtue of the neighborhood knowledge of training examples. The degree of dispersion of the continuous labels in the *y* space was also one of the influencing factors of the transductive model. For prediction, we selected the epsilon-support vector regression (e-SVR) to construct the transductive model. The non-linear real-time features were extracted using a combination of wavelet packet decomposition and bi-spectrum analysis. The performance of the proposed approach was evaluated using the DEAP dataset and Stroop training. The results indicated the effectiveness of the transductive model, which had a better prediction performance compared to traditional methods. Furthermore, the real-time interactive experiment was conducted in field studies to explore the usability of the proposed framework.

## 1. Introduction

As a psychological–physiological process, stress is the direct reflection of the conscious or unconscious human object or situation [1,2]. Physiological signals can better reflect the real-time stress state compared to facial expressions [3]. Stress recognition based on physiological signals allows researchers to directly assess the user’s internal state, making it a key element of the human-to-computer interaction. State-of-the-art sensor technology makes it possible to monitor various physiological signals to analyze human behavior, cognitive science, social psychology, and group perception [4,5,6]. Wearable sensor technology to monitor a specific person’s stress recognition can specifically serve medical and mental health purposes [7], as well as the accompanying care services. Some physiological signals derived from wearable non-invasive devices, such as the galvanic skin response (GSR), respiratory rate, electromyography, and blood volume pulse (BVP) can detect the emotional state of the body [8], making real-time stress monitoring for humanity a reality [9]. The wearable sensors for stress recognition are divided into two major types: Biochemical sensing [10,11] and Physical sensing [12,13]. Biochemical sensing has the capacity to monitor clinically relevant biomarkers, such as the cortisol from sweat, using electrochemical technology. Different from the former, physical sensing detects the surroundings, such as pressure, conductance (such as GSR), light (such as BVP), torsion, and temperature.

External stimuli and emotional events can unconsciously stimulate the activity of the autonomic nervous system (ANS), further linking the brainstem and spinal cord to the internal organs for the regulation of physiological parameters. BVP is a measure of the peripheral blood vessel volume and is commonly obtained using photoplethysmography (PPG). PPG usually consists of light emitters and detectors. Measuring the reflected light on the skin is an indicator of the blood volume in the peripheral blood vessels. Under stress conditions, the effects of epinephrine on the human left ventricle can be estimated effectively by the BVP over a given period [14]. It has been proven that an increase in stress will lead to a decrease in the BVP [15]. GSR is a measure of the skin resistance (or conductance). The conductance of the skin changes with the activity of the sweat glands, which are controlled by the sympathetic nervous system. Skin conductance increases under stress due to the increase in moisture on the surface of the skin, leading to an increase in the flow of electricity [16]. Therefore, BVP and GSR signals were chosen for stress recognition in this paper. For the sensors, we selected the classic wearable wireless multisensory device branded as Empatica, based on the physical sensing released by Picard’s team [17]. The device provides high-resolution data quality using the proprietary artifact removal technique.

However, there are still some challenges such as the differences in individual physiological signals [18,19]. These differences exist not only between people, but also between individuals at different background levels, i.e., each humanoid has specific body conditions which cause diverse physiological signals. Meanwhile, due to the difficulties in labeling the physiological signals, the dataset extracted from an individual is always composed of dispersed data and imbalanced samples that cause the poor recognition effect.

**Figure 1:**
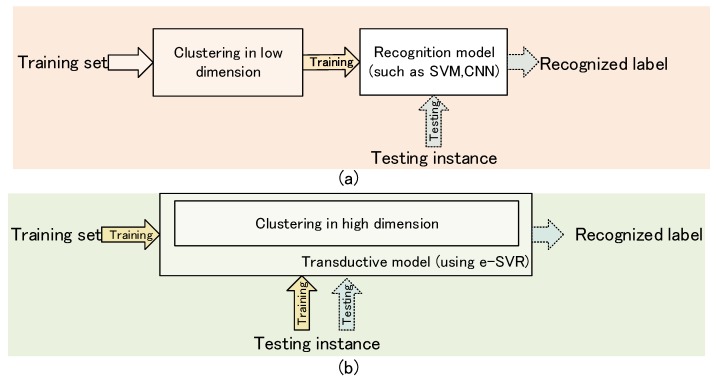
Comparison of the recent study and the proposed transductive model: (**a**) The flow diagram of the recent study, including the two main processes; (**b**) The flow diagram of the transductive model.

A recent study on personalized stress or emotion recognition, based on physiological signals, involved two main processes [20,21,22], as shown in Figure 1a. The first process filtered out the distinguishable data through feature processing and clustering. The second process focused on the establishment of the training models, such as the Support Vector Machines (SVM), using the above distinguishable data. The literature has shown that such processes, especially in the first process of clustering, can be beneficial for the performance of recognition. However, there are still two problems as follows:

(1) For the first process, few recent efforts have measured the relevant data in high dimensions, but rather they have depended on the clustering before training. This means that the correlation measurement is performed in the low-dimensional space, whilst the linear prediction is performed in the high-dimensional space, which may cause the hyperplane to be affected by unfiltered outliers. Regarding the support vector regression (SVR), the outliers, meaning the irrelevant data, may lead to serious consequences [23].
(2) For the second process, these studies rely on the widely used inductive reasoning, based on global optimization, and they only consider processing the training set instead of the test instances. Inductive reasoning is based on learning from experience, which aims to generate meanings from the training set to identify patterns and relationships to build a recognition model. It is useful for a global model of the approximate problem, but for each individual case, it does not have good generalization ability.

In order to address the above issues, a transductive model is proposed by combining transductive learning with clustering methods based on neighborhood knowledge in high dimension, as shown in Figure 1b. Specifically, the neighborhood knowledge refers to the continuous labels and the similarity measure in the feature space of nearby examples. Unlike the existing research, the clustering process was performed in the recognition model. Compared to inductive reasoning, the transductive learning [24], as the theoretical basis of the proposed transductive model, was more suitable for application, because it did not focus on the overall precision of the model but rather each individual case [25]. This satisfied the demand of physiological signals-based stress recognition. In the transductive learning, the test instances and training set interacted to determine the optimal subset, by which a particular local regressor can be constructed. The aim of the transductive model in this paper was not only the clustering but also the outliers’ exclusion, and finally to reduce the errors caused by the individual differences to improve the performance of the stress recognition. The transductive model adopted the epsilon-Support Vector Regression (e-SVR) to perform the prediction, which was divided into the specific transductive-SVR (ST-SVR) and the transductive-SVR (T-SVR), according to the source of the training set. In addition, the inherent homogeneity of the stress states of different individuals was also considered, and the prediction performance of the T-SVR (samples from all subjects) was compared to the performance of the ST-SVR (samples from one individual subject).

**Figure 2:**
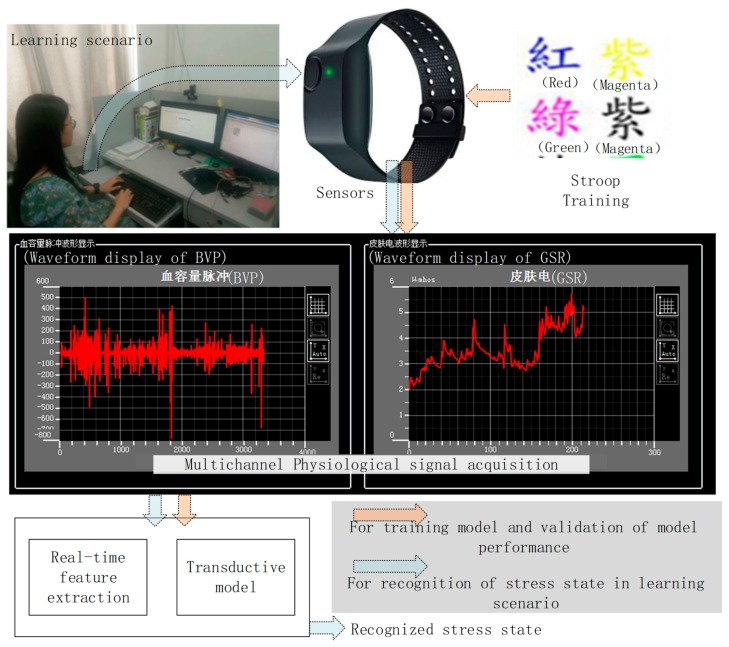
Block diagram of the proposed stress recognition in the learning scenario.

We also provided a framework for field studies in the learning scenario to explore the usability of the proposed model. The academic pressure and frustration, etc., of students lead to depression and anxiety, and therefore, it is necessary to adjust the teaching content or to provide psychological counseling based on the mental stress of the students. In our study, using the computerized approach of the interactive interface for learning English words, we established a real-time framework that could recognize the student’s stress state using peripheral physiological signals as shown in Figure 2. A self-established dataset was collected based on the Stroop Effect [26], with concurrent labels to train the recognition model. The main contributions of this work are fourfold:

(1) We proposed a transductive model to reduce the generalization error for stress recognition from the peripheral physiological signals;
(2) Extracting the non-linear peripheral physiological features, which can characterize the time-frequency features of small sliding windows to better support real-time stress recognition;
(3) The performance of the proposed method was investigated on the self-established database, as well as the widely used DEAP dataset [27]. The results indicated that our method outperformed the existing methods.
(4) The real interactive experiment was performed to explore the feasibility of this framework based on non-invasive sensors.

The rest of the paper is organized as follows: First we describe the related work in Section 2. Data collection and assessment methodology are presented in Section 3. The methodologies, including the peripheral physiological feature extraction and the stress recognition method, are presented in Section 4. We present the results in Section 5 and the discussion in Section 6. Finally, we make a conclusion of our work in Section 7.

## 2. Related Work

### 2.1. Sensor-based Physiological Signals

Physiological signals through wearable devices include the electroencephalography (EEG), electrocardiography (ECG), GSR, BVP, heart rate (HR), and derived blood oxidation. The acquisition method of physiological signals generally depends on the specific sensors. Rahim et al. [28] designed the non-contact wearable EEG sensors, which consisted of a 5.5 × 3 cm^2^ sized hardware and four stainless steel electrodes integrated into a regular sport hat. Murat et al. [29] reported a fully-wearable medical garment for the electrocardiogram (ECG) recording, instead of using sticky, pre-gelled electrodes. Ammar et al. [30] invented a system for the detection of low blood oxygen. In our paper, the peripheral physiological signals were recorded using the Empatica E4 for stress recognition. The details of the wristband were described in Appendix A. The advantages included its flexibility and low latency, which were more suitable for real life use.

### 2.2. Transductive Learning

Transductive learning was first proposed by Vladimir in the 1990s, with the aim of creating a model that was more applicable to the problem domain compared to a more general model. Haibin et al. proposed the localized support vector machine using the cluster method named MagKmeans [31]. Pang et al. [25] built a unique local model for the classification of each test sample. The approach had two steps: Neighbor-sample filtering and Regional decision making. The samples near the test sample were extracted for training, similar to the work of Pang et al. In recent years, there have been many optimization problems for transduction learning on SVMs. For example, Hakan et al. proposed a robust and fast transductive method applicable for large-scale data [32]. Yanchao et al. utilized the concave–convex procedure and the variation of stochastic variance reduced gradient methods to solve the non-convex optimization problem of transductive-SVMs [33]. In almost all the studies, transductive support vector machines were classification models, and it was considered to extract training samples close to the test samples with the same discrete categories. Moreover, they are aimed at optimizing the objective function. In this paper, we reconstructed the objective function of the cluster to construct the transductive mode of the continuous label and regression models.

### 2.3. Stress Recognition

Physiological signals can provide information regarding the intensity and quality of an individual’s internal state [34]. There are many researches about the multimodal method for stress recognition, and almost all of them consider the physiological signals. For example, Bosun et al. [35] developed the Deep ECGNet for monitoring mental stress based on the ECG waveforms. Youngjun et al. [36] proposed a DeepBreath model based on the Convolution Neural Network (CNN), reaching an 84.59% accuracy in discriminating between two levels of stress, and a 56.52% accuracy in discriminating between three levels of stress. The authors’ major technical contribution was the acquisition of two-dimensional respiration variability spectrogram (RVS) sequences. Wei et al. [37] used electroencephalography (EEG) signals to recognize the stress state of crew members using deep learning techniques as well. Moreover, some researches also focused on the facial information correlated to stress, where, for example, Giannakakis et al. [38] developed a framework for the detection and analysis of stress/anxiety emotional states using video-recorded facial cues. It also indirectly proves that facial expressions are related to the stress state. These studies have made some achievements using deep learning; however, it would not be suitable for use in real-time or short-interval stress recognition based on mobile or wearable devices, due to the time and equipment costs. In addition, deep learning is mostly being used with the inductive method.

Additionally, there are some researches based on machine learning, where Oscar et al. [39] proposed a machine learning approach combining two sensor systems for use in stress detection, which achieved a 0.67 accuracy. It claimed that the results were affected by the large between-subject variances to stress. Lucio et al. [40] focused on the real-time detection of mental stress and achieved an 89.8% classification accuracy for the stress state and a 74% classification accuracy for the relaxation state, using the k-nearest neighbors algorithm (K-NN).

However, there are still some shortcomings, for example, there are limited classification levels of stress [41] and a low generalization ability on physiological signals. To address these issues, the transductive model using the SVR was presented to recognize the continuous labels of stress.

## 3. Data Collection and Assessment Methodology

We followed two main experiments of the framework as shown in Figure 2. In the first experiment, Stroop training was performed for real-time data collection to train the model for field studies. Concurrent labels were automatically marked. In the second experiment, the trained model was used to recognize the subjects’ stress state in the learning scenario. Retrospective assessment was provided for the validation of this framework. For each subject, the whole process took 2 h. We paid the reward-related participation fees to guarantee the engagement of the subjects in the second experiment.

### 3.1. Participants

The subjects comprised 20 postgraduate students from the University of Science and Technology Beijing, with an age range from 24 to 30 years, Mean (M) = 25.05, Standard Deviation (SD) = 1.53. Before taking part in the study, all the students had received more than six years of formal EFL (English as a foreign language) education and had passed the EFL test of the National College Entrance Examination in China.

### 3.2. Stroop Training

**Figure 3:**
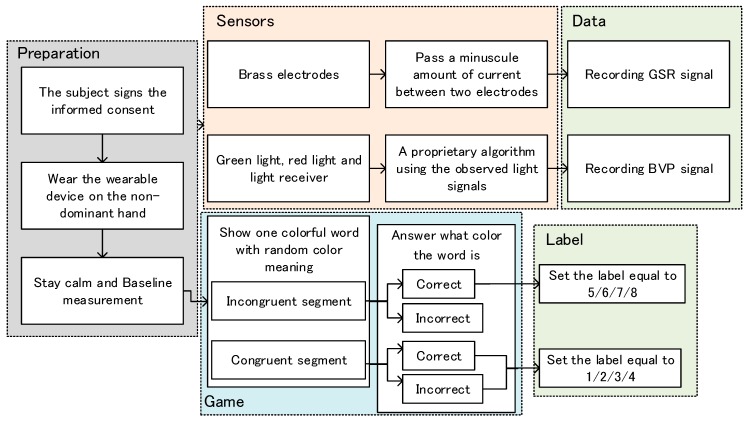
Block diagram of the Stroop Training.

The “Stroop Effect” has proven the ability to inspire human mental stress, and there are many existing computerized interactive experiments based on this theory, such as in [42]. The interactive Stroop training was performed using peripheral physiological signals with concurrent labels, conducted on a personal computer (PC). The detailed experimental process was as shown in Figure 3.

The detailed steps were as follows:

(1) The subjects signed the consent forms.
(2) The subjects wore the wearable device on the non-dominant wrist, for recording the GSR and BVP signals simultaneously. The technical documentation [43] from Emaptica was strictly followed for recording the signals effectively.
(3) The subjects were asked to stay calm for a period of the baseline measurement.
(4) After the game started, the PC screen displayed a word, the meaning of which was one kind of color, and its font color was random.
(5) Subjects were asked to select the correct key that represented the font color instead of the word meaning. They would wait 2 seconds and then the next word was taken.
(6) The difficulty of the game ranged from simple to difficult, with four levels. Each level has 40 words. The game at the simple level waited longer for the answers, and vice versa.
(7) When the answer time was exceeded, the next word was taken, and the previous word was treated as an incorrect answer.

**Figure 4:**
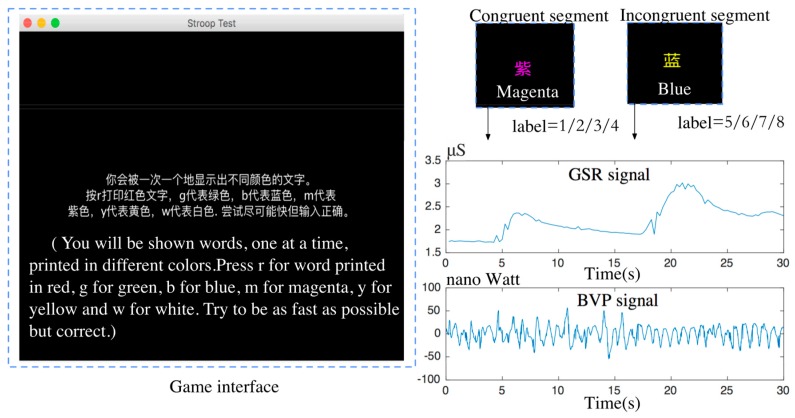
The process of congruent labels.

The moment when each event occurred was recorded synchronously to help set the concurrent labels. Figure 4 shows the labeling process. The automatic labels’ definition was based on the difficulty level of the game and the consistency between the font color and word meaning. The congruent segment had a matching meaning and font color in the words, whilst the incongruent segment had a mismatched meaning and font color in the words. During the incongruent segments, the stress state presented by sympathetic activation might cause potential concurrent changes [44]. The period from the new incongruent segment shown to one key pressed, was set to the stress label (5/6/7/8). Shaomei et al. [45] found that stress increased significantly with the increasing difficulty of the game.

Therefore, in the simple mode, we set the label equal to 1 during the congruent segment, and the label equal to 5 during incongruent segment, which was incremented respectively according to the difficulty level. For the high stress state, only the epoch of the correct answers was labelled, which ensured that the real stress states of the subjects were elicited under the conditions of engagement.

### 3.3. Learning Scenario

**Figure 5:**
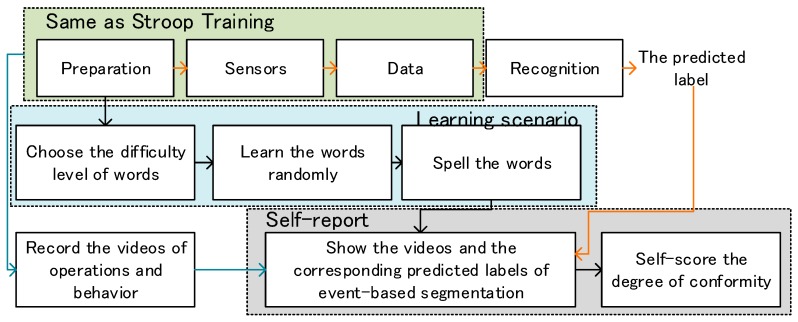
Block diagram of the experiment in the learning scenario. The three lines mean three concurrent processing.

This experiment was performed using a wrist strap and a PC with one keyboard. Before the experiment started, the subjects were asked to keep calm and to keep their non-dominant hand still for baseline recording. Figure 5 shows the detailed processing.

(1) The processes of preparation, sensors and data acquisition were the same as Stroop training.
(2) There are three processes involved in learning scenario as shown in Figure 5. The order of words set randomly in advance was the same for each subject in each process.
(3) The words without meaning were shown successively and the subjects selected the difficulty level of each word quickly.
(4) The subjects learned each word with meaning within one minute. And each subject need to learn 60 words.
(5) The interactive interface only showed the meaning of each word and the subjects were required to spell the words within a limited time. The interactive interface is shown in Appendix B.

Self-report was taken to measure the reliability of recognized stress using the approach of retrospective assessment. Subjects watched their own video of operations and behavior, including their facial expression and upper body postures, to score the degree of conformity (CD). Since the retrospective assessment does not guarantee accuracy per second, we chose fragmented scoring based on event-based segmentation [46]. Specifically, each event, such as operating the keyboard or a minor change of expression or body posture, was segmented as an event-based segmentation. Each event-based segmentation contained two kinds: faces and operations, placed together as a group. Each group segmentation was displayed in the order in which the events occurred, and the predicted pressure value curve for that segmentation was also displayed synchronously. The scoring of the CD was assessed by the subjects themselves and ranged from 0 to 100.

Videos of the behavior and operations were recorded using two HD 720P camera sensors (Sony, Tokyo, Japan). One camera faced the subject’s face and the other camera was placed behind the subject to ensure that the subject’s operation and PC screen were recorded. Video was recorded at 30 fps, in 24-bit RGB with three channels, 8 bits/channel, with a resolution of 640 × 480 pixels.

## 4. The Stress Recognition Methodology

### 4.1. Peripheral Physiological Features

In the experiments, the peripheral physiological data (BVP and GSR) of the subjects were measured simultaneously using a wearable device (the Empatica from Picard’s team). BVP signals were recorded with a sampling rate of 128 Hz by measuring the reflected green and red light-emitting diodes (LEDs). GSR signals were recorded with a sampling rate of 4 Hz by placing two electrodes on the wrist.

#### 4.1.1. BVP Signals

**Figure 6:**
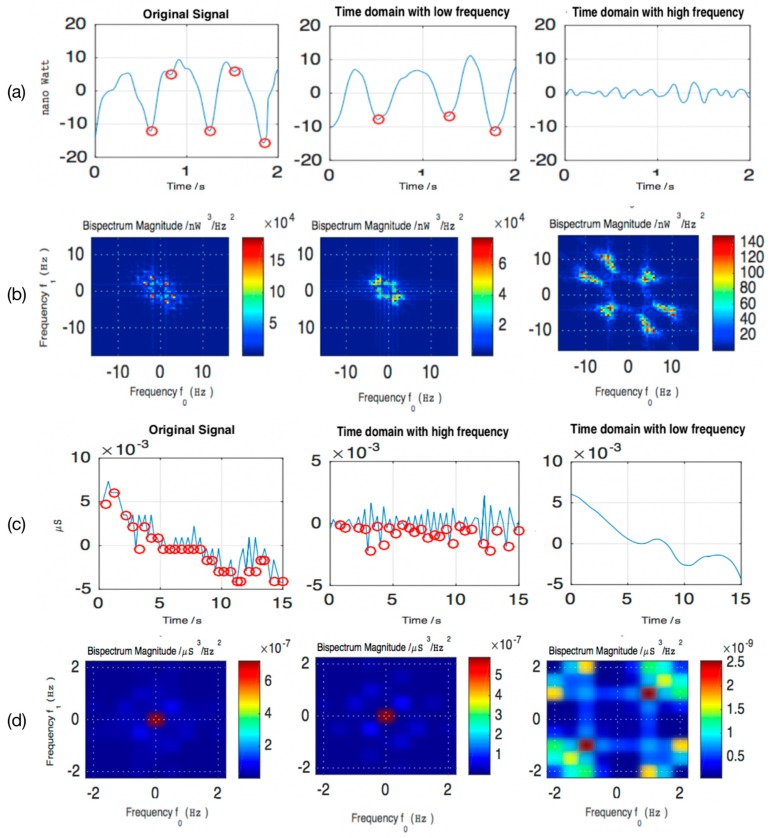
The examples of morphological wavelet packet-bi-spectrum analysis from the BVP and GSR: (**a**) The signal decomposition of BVP; (**b**) Bispectrum magnitude of BVP; (**c**) The signal decomposition of GSR; (**d**) Bispectrum magnitude of GSR.

Similar to the ECG, BVP signals are composed of wave events, as shown in Figure 6a. As the blood volume changes with the pulse, the IBI (inter-beat interval) can be detected from the BVP signals, so that the heart rate (HR) and heart rate variability (HRV) related characteristics can be calculated. Diastolic point is the local minimum of the BVP used in the IBI computation. However, the presence of the dicrotic notch causes the diastolic point to be inaccurately obtained by the extreme values calculation (EVC).

#### 4.1.2. GSR Signals

The GSR signals include tonic (slow) and phase (rapid and stimulus-related) activities associated with emotional arousal. Phasic activity can be treated as an evoked discontinuous response to external stimulus. Tonic activity is often referred to the background level of a particular physiological measure. Therefore, in the case of external stimulation, the GSR signals of a time period include not only the intermittent pulse signal (related to the excitation), but also the slowly varying smooth signal caused by factors such as hydration and autonomous regulation. Skin conductance response (SCR) relates to the former and its peak is the important indicator characterizing the instantaneous stress state. There is usually baseline drift a phenomenon. Thus, feature extraction should be considered to avoid the impact of baseline drift.

#### 4.1.3. Extracted Features

Similar to the BVP, the extremum of the original GSR signals cannot be used to calculate the current features. Real-time stress recognition requires high time resolution, so the sliding window should not be set too wide. However, a narrow sliding window may result in poor frequency resolution, which is not conducive to spectrum analysis. Therefore, in the first process, we used wavelet packet decomposition to obtain the low frequency and high frequency signals of both the BVP and GSR, as shown in Figure 6a,c. Amongst them, due to the different sampling frequency, two second windows could show the integral two or three fluctuations in the BVP, while in the GSR, the fifteen-second window size was selected to show the tonic activities. The advantage of using wavelet transform is to analyze non-stationary signals with obvious time-frequency local changes, which can effectively enhance the transient information hidden in the physiological signals. For the BVP signal, the low frequency signal better extracted the signal containing only the diastolic point and the contraction point, e.g., the red point in the time domain with low frequency in Figure 6a. For the GSR signal, the high frequency signal represented the rapid change of the SCR. Therefore, in the second process, using EVC, the peaks of the BVP could be obtained in low frequency and the peaks of the GSR could be obtained in the high and low frequency, respectively. In addition, the phase was used to determine the relative progress of one oscillatory cycle at a particular time. Owing to the translation invariant feature of the spectral amplitude, the lack of phase information increases the convergence between the heterogeneous targets, resulting in a decrease in the regression performance. Therefore, in the third process, we used bi-spectrum analysis to characterize its spectral features, as shown in Figure 6b,d. Finally, the list of extracted features and their descriptions are given in Table 1. The extracted features of GSR are divided into two types: high frequency and low frequency. Most of these features are not affected by baseline drift, except for the low-frequency high-order cumulants. And normalization we used can reduce the impact of baseline drift to the low-frequency high-order cumulants. For the DEAP dataset, the method of extracting the features was the same and only the sliding window size was changed.

### 4.2. Transductive Model

For the e-SVR, only the example points outside of the ε pipe wall and on the ε pipe contributed to the solution, which was called the support vector. The complexity of the SV function representation is independent of the dimension of the input space $X_{tr}$, and it depends only on the number of SVs. The kernel trick projects the input features into a higher-dimension feature space and it helps to construct the hyperplane. The original convex optimization problem is written as:

(1) $$\begin{array}{c} \min\;\frac{1}{2}{\left\|\omega \right\|}^{2}+C\overset{L}{\underset{i=1}{\sum }}(\xi_{i}+\xi_{i}^{*})\\ s.\;t.\;\{\begin{array}{c} y_{i}-b-\langle \omega ,x_{i}\rangle \leq \varepsilon +\xi_{i}\\ y_{i}-b-\langle \omega ,x_{i}\rangle \geq \varepsilon +\xi_{i}^{*}\\ \xi_{i},\xi_{i}^{*}\geq 0 \end{array} \end{array}$$

We built a transductive model combined with the optimization problem in the SVR, for local generalization. Therefore, the optimization problem was rebuilt as follows:

(2) $$\begin{array}{c} \min\;\frac{1}{2}{\left\|\omega \right\|}^{2}+C\overset{L}{\underset{i=1}{\sum }}\Omega \left(X_{te},x_{i}\right)(\xi_{i}+\xi_{i}^{*})\\ s.\;t.\;\{\begin{array}{c} y_{i}-b-\langle \omega ,x_{i}\rangle \leq \varepsilon +\xi_{i}\\ y_{i}-b-\langle \omega ,x_{i}\rangle \geq \varepsilon +\xi_{i}^{*}\\ \xi_{i},\xi_{i}^{*}\geq 0 \end{array} \end{array}$$

where $\xi_{i}$ and $\xi_{i}^{*}$ are slack variables, and $\Omega \left(X_{te},x_{i}\right)$ is a weight function used to measure the similarity between each example and test example in the high-dimensional feature space and y-space. The first term relates to the support vectors which weight the margin. The other is the penalty which determines the trade-off between the flatness of the regression function, and it causes the stronger influence on neighborhood rather than the far away support vectors.

Equation (2) can be transformed into a dual optimization problem:

(3) $$\begin{array}{c} \max\;\overset{L}{\underset{i=1}{\sum }}y_{i}\beta_{i}-\frac{1}{2}\overset{L}{\underset{i,j=1}{\sum }}\beta_{i}\beta_{j}\Phi \left(x_{i},x_{j}\right)-\varepsilon \overset{L}{\underset{i,j=1}{\sum }}\beta_{i}^{*}\\ s.\;t.\;\{\begin{array}{c} \sum_{i=1}^{L}\beta_{i}=0\\ \beta_{i}=\alpha_{i}-\alpha_{i}^{*}\\ \beta_{i}^{*}=\alpha_{i}+\alpha_{i}^{*}\\ \alpha_{i},\alpha_{i}^{*}\in \left[0,C\Omega \left(X_{te},x_{i}\right)\right] \end{array} \end{array}$$

where $\alpha_{i}$ and $\alpha_{i}^{*}$ are the LaGrange multipliers affected by $C\Omega \left(X_{te},x_{i}\right)$, which determines their upper bound. As a result, the transductive model leads to the local SVR model.

Considering the similarity in the high dimension, in the transductive SVR method, the clustering method was included to extract the support vectors in the high-dimension space using neighborhood knowledge. The infinite-dimensional feature vector could be transformed into a finitely computable value based on the property of the kernel function. The square norm of the difference between two feature vectors Φ(·) represents the distance between them in the high dimension:

(4) $$\begin{array}{c} \kappa_{i,j}=\|\Phi \left(x_{i}\right)-\Phi \left(z_{j}\right)\|=\sqrt{\langle \Phi \left(x_{i}\right)-\Phi \left(z_{j}\right),\Phi \left(x_{i}\right)-\Phi \left(z_{j}\right)\rangle }\\ =\sqrt{K_{i,i}^{tr}-2K_{i,j}+K_{j,j}^{te}} \end{array}$$

where $z_{j}\in X_{te}$ and $x_{i}\in X_{tr}$. The $\kappa_{i,j}$ characterizes the degree of similarity between the test instance and the training instance. $K_{i,i}^{tr}$ and $K_{j,j}^{te}$ are the kernel matrix of the training set and the test set, respectively. $K_{i,j}$ can be written as:

(5) $$K_{i,j}=\left[\begin{array}{ccc} k\left(x_{1},z_{1}\right) & \ldots & k\left(x_{1},z_{M}\right)\\ \vdots & \ddots & \vdots \\ k\left(x_{L},z_{1}\right) & \ldots & k\left(x_{L},z_{M}\right) \end{array}\right]$$

The emotional type of label is generally subjective rather than objective, and therefore, similar examples do not necessarily have similar corresponding labels. A very small number of mislabeled outlier examples can cause large errors in the support vector machine, because the SVMs are designed to use the structural risk minimization principle. At the same time, in the high-dimensional space, the error-labeled outlier examples behave differently from the members with similar labels. In addition to considering the degree of similarity among the examples, we considered the degree of dispersion of the continuous labels in the y space. The clustering criterion was modified to ensure that the extracted cluster contained training examples without outliers from y. At the same time, the arrangement of the outliers is not always radical, but it may be transitional, so continuous values are provided as a reference for combinatorial selection, and the objective function is set to:

(6) $$\underset{\sigma_{i}}{\min}\overset{M}{\underset{j=1}{\sum }}\overset{N}{\underset{i=1}{\sum }}\kappa_{\sigma_{i},j}+\rho \overset{M}{\underset{j=1}{\sum }}\overset{N}{\underset{i=1}{\sum }}\frac{\left|y_{\sigma_{i}}-\mathrm{median}\left(y\right)\right|}{\mathrm{mad}\left(y\right)}$$

where ρ is a scaling parameter. The Stahel–Donoho estimator [48] is used to measure the similarity in the y-space. median(y) is a robust univariate estimator of location, while mad(y) is a univariate estimator of spread. In the objective function, the first term is a cluster cohesion measure in the high dimension, whilst the other term measures the skewness of the continuous labels’ distributions in each cluster. Minimizing the former would ensure that the training examples are close to the test examples, while minimizing the latter would give rise to highly correlated similarity amongst the training examples in the *y* space.

Therefore, we can construct the lower rank matrix with the element $\Omega \left(X_{te},x_{i}\right)$ by setting:

(7) $$\Omega \left(X_{te},x_{i}\right)=\{\begin{array}{cc} 1 & x_{i}\in \left\{x_{\sigma_{1}},\ldots ,x_{\sigma_{N}}\right\}\cap X_{tr}\\ 0 & \mathrm{otherwise} \end{array}$$

## 5. Results

The verified results are based on two following parts: Prediction performance, namely recognition performance, of the proposed transductive model, and the field study in learning scenario.

### 5.1. Transdutive model

#### 5.1.1. Overview

##### Benchmark

The DEAP dataset is a multimodal dataset that was created for the research of human affective states. There were 32 subjects whose peripheral physiological signals were recorded as each subject watched 40 one-minute highlight music videos. Those participants performed a self-assessment in terms of their levels of arousal, valance, etc., which were continuous values. In the DEAP dataset, two peripheral physiological signal channels (BVP, GSR) are utilized for features extraction, which ensures a comparable benchmark. Theorists found that stress was a negative valence, which meant that the stress state was comparable to the valence with a certain degree. For example, Catherine et al. [49] found that increased stress reactivity was associated with a stronger negativity bias of valence by measuring the salivary cortisol. Alexandros et al. [50] utilized the self-report and found a high correlation (85%) between stress and negative valence. Therefore, the valence from the DEAP dataset was used for the labels.

##### Data Distribution

**Figure 7:**
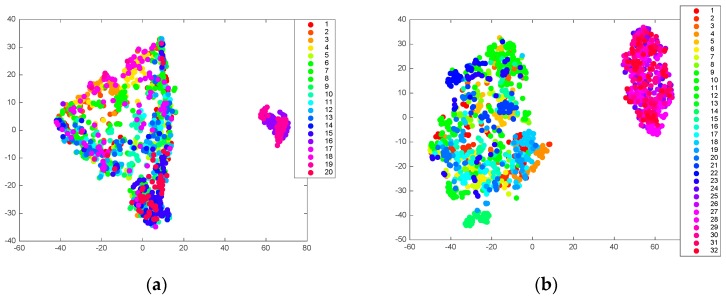
Visualization of the high-dimensional features using t-SNE. Each class represents the specific subject ID: (**a**) From Stroop training; (**b**) From the DEAP dataset.

The model for prediction was evaluated in the datasets that allowed the inclusion of peripheral physiological signals and considered the diversity of the subjects. Figure 7 shows the inter-class dispersed distribution of the Stroop training and DEAP dataset using t-distributed stochastic neighbor embedding (t-SNE) [51], with a fixed perplexity 40. It reflected the obvious individual differences in these datasets. Factors such as gender, age, and specific body conditions, were among the individual differences that adversely affected classifier performance in the previous works [52,53]. In our work, the peripheral physiological features were extracted to represent the physiological differences caused by these factors [54]. In addition, there were different types in the within-class distribution, i.e., some were collected while some were dispersed.

##### Compared models

**Figure 8:**
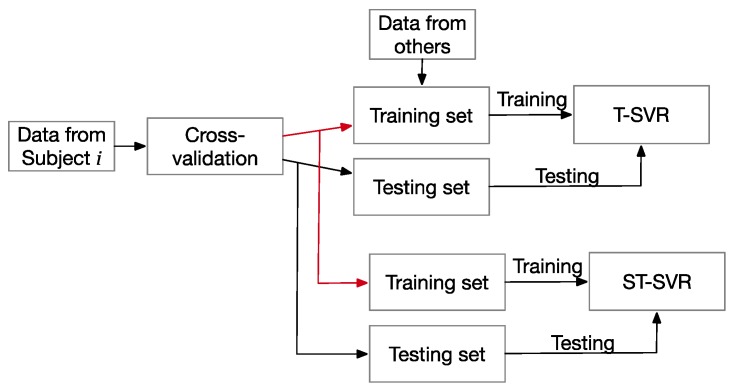
The training process of T-SVR and ST-SVR.

To evaluate the effectiveness of the proposed method, the paper provides the epsilon-SVR (e-SVR), the linear regression (LR), the CNN for Regression, the ST-SVR, and the T-SVR, on a workstation using MATLAB and the SVM library, LIBSVM as in Reference [55]. The labels and features were normalized. The training details were as follows:

- e-SVR and LR: Due to the dispersed data distribution, training the data from all subjects could not reach the state of convergence. Therefore, the data from one subject was used to train one model.
- CNN: We used an individual subject for training the CNN using the original GSR and BVP input. Like the EEG signal for prediction using CNN [56], the GSR and BVP could be treated as two channels from the EEG for independent CNN training, and then the two outputs were concatenated to input the fully connected layer. Moreover, the output layer’s activation function was a linear function, as in Reference [57].
- ST-SVR: This differentiates the categories for training. As shown in Figure 8, the ST-SVR uses each subject’s individual examples to train the models. The training set and the test set of the subject *i* is divided by the cross-validation from subject *i*’s dataset.
- T-SVR: Involves training without distinction. For subject *i*, the T-SVR uses all the subjects’ examples to train the model, except the testing set from subject *i*.

##### Hyperparameters

Before formal training, cross-validation was used to find the optimal hyperparameters for each model. For the SVR based models, the search range was set to $\left[2^{-8},2^{8}\right]$, with a step size of 0.5. Because overfitting and underfitting can cause excessive test error, the test error in cross-validation, as the criteria for the choice of N, measures the case of overfitting and underfitting. We fixed *N* = 5 in the proposed T-SVR and ST-SVR through cross-validation. Because we found the results of test error to be stable across a similar choice of the *N* value. In addition, a too large *N* value will cause underfitting, whilst a too small *N* value will lead to overfitting.

We applied these models using an Intel Core i7 2.6GHz, 16GB RAM, and 64bit macOS 10.13.6 system. Moreover, the stress recognition in the learning scenario was provided for real-time automatic application and exploration of the relationship between stress and academic difficulty, etc.

#### 5.1.2. Stroop Training Results

The models that were built are presented in Table 2. The mean absolute error (MAE) was used to measure the performance of these models, which was defined as follows:

(8) $$MAE=\frac{1}{l}\overset{l}{\underset{i=1}{\sum }}\left|y_{i}-{\hat{y}}_{i}\right|$$

A group of the absolute error of each model was obtained from all the subjects. We computed the mean, e.g., MAE, and the SD for each model. Among them, the SD was used to measure stability of the absolute error. Since there was no clear time node between the test and training in transductive learning, a training process and a test process were used for the time calculation for each model.

Several key conclusions could be drawn from Table 2. Firstly, it prioritized the performance of prediction as follows: ST-SVR, T-SVR, CNN, e-SVR, and LR, though the difference between ST-SVR and T-SVR was quite muted. Secondly, the time performance could be ordered as: LR < e-SVR < ST-SVR < T-SVR < CNN. Third, the better the performance of the prediction or time, the smaller the SD. Fourth, the proposed models had the better performance of the model, but the time consumption was not as good as e-SVR and LR. Fifth, although the T-SVR was based on all the subjects’ data, the performance of T-SVR was better than all the models except the ST-SVR, which was based on the subject-dependent training.

#### 5.1.3. DEAP Dataset Results

**Figure 9:**
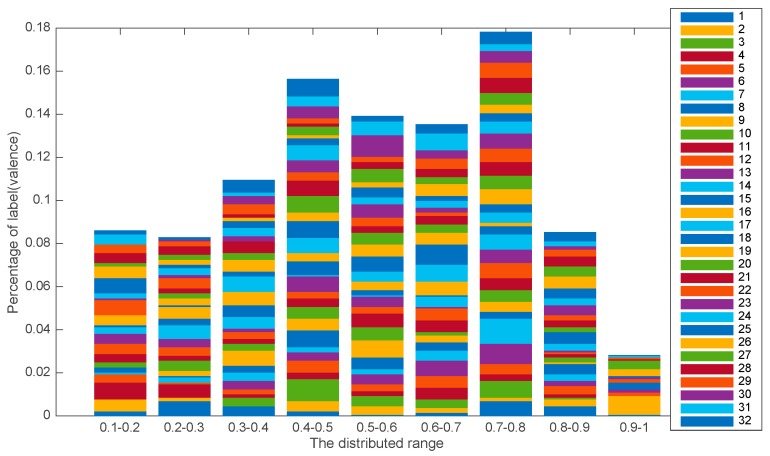
The label distribution of the DEAP (valence). Each legend represents the number of subjects.

Before delving into the results, there is an issue to be discussed regarding the DEAP dataset. Figure 9 shows the label distribution (valence) of the DEAP, where we found that there was a sample imbalance problem in the DEAP dataset for each subject.

Table 3 shows an overview of the performance of each model. Similar to Stroop training, ST-SVR had the better performance of prediction, though its MAE was similar to the T-SVR. The performance in prediction could be ordered as: ST-SVR, T-SVR, CNN, e-SVR, and LR. However, in the DEAP, e-SVR achieved the best time performance. LR and e-SVR still took much less time than the proposed models and the CNN.

#### 5.1.4. Comparison of Stroop Training and DEAP Dataset Results

Similar to Stroop training, we considered the prediction performance in the DEAP. Owing to the existence of games at different levels of difficulty, Stroop training had no serious sample imbalance problem, unlike the DEAP. Therefore, it could be intuitively concluded that the Stroop training performance was better than the DEAP. However, by comparing Table 2 with Table 3, we found a contrary conclusion. In addition, the training time of the DEAP was less than the Stroop training, and the model of the best time performance was different. DEAP and Stroop training have a similar ranking of the prediction performance. ST-SVR does the best work whether using DEAP (MAE = 0.156, SD = 0.043) or Stroop training (MAE = 0.193, SD = 0.069).

#### 5.1.5. Comparison to State-of-the-Art Results

The work by Martinez et al. [58] perhaps represents the state-of-the-art in real-time stress recognition using non-invasive physiological sensors. The authors developed a system that classified stress in three states: “Low Stress (LS)”, “Medium Stress (MS)”, and “High Stress (HS)”. The GSR and HRV signals were used for feature extraction. The classification model was the Finite State Machine (FSM) based on quantitative analysis (QA). Finally, the authors’ best subject-dependent model achieved an F1 score between 0.943 and 0.970 and 0.984.

**Figure 10:**
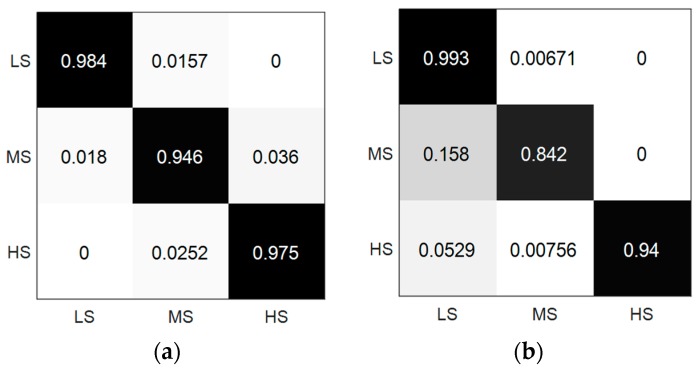
The Confusion matrix of the ST-SVR and T-SVR: (**a**) ST-SVR; (**b**) T-SVR.

For comparison, we presented the classification results by dividing the label and the regression results into three equal intervals. The performances of the ST-SVR and T-SVR were used to compare with the authors’ method. Table 4 describes the F1 scores for LS (0–0.33), MS (0.33–0.66), and HS (0.66–1). We achieved a better performance for LS, except for MS and HS, and the ST-SVR attained a slightly higher mean of the F1 score (0.968) compared to the authors’ method (0.965). However, it is difficult to assert that our methods are an improvement over the previous state-of-the-art methods, since their sliding window size was set from 20 to 35 seconds, which was far longer than our 3 seconds. The datasets differ in terms of both the sensors and the method of annotation could cause the fuzzy bounds of comparison. Moreover, the confusion matrix of the ST-SVR and T-SVR is shown in Figure 10. In both ST-SVR and T-SVR, the smallest recall and F1 score exist in MS, but the biggest recall are in LS. Although T-SVR has the greater recall of LS than ST-SVR, the F1 score of LS in T-SVR is smaller than in ST-SVR due to the low precision.

### 5.2. Learning Scenario

For the field studies, we presented the real-time interactive experiment in the learning scenario. We set the recognition rate at 1 s per time, with a 3 s sliding window, which meant that the last 3 seconds of the signal represented as one epoch, was selected for each recognition. The trained T-SVR was used to predict stress.CD was used to measure the reliability of stress recognition using a retrospective assessment.

**Figure 11:**
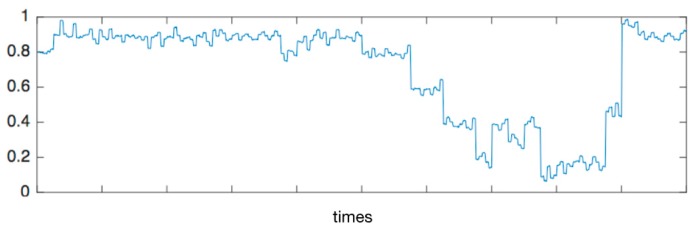
The example of the predicted stress state value from Subject 7th.

Over the whole process, the answering time, recognized stress value, score of spelling, and the standard of question asked were recorded for analysis, as shown in Table 5. The mean of the answering time for difficult questions was higher than the one for easy questions. However, they both had a high standard, with skewed distribution. Owing to real-time stress recognition, there were generally different predicted values and minor delays during the answering of one question as shown in Figure 11. Therefore, we chose the median of the most similar values to indicate the stress state at that stage and divided it into a low stress state and high stress state by comparison with the cut-off of 0.5. Two conclusions could be drawn in the table:

(1) Both the difficult and easy questions had a similar mean and similar SD of the CD. It indicated the reliability of the real-time stress recognition.
(2) The ratio of the number of high stress to the number of low stress was 1.79 during the difficult questions period, whilst the ratio was 0.52 during the easy questions period. It could be concluded that the difficult questions led to more stress.

## 6. Discussion

The pervasiveness of wearable sensors, such as the Empatica, opens new opportunities in the design of real-time stress or emotional state monitoring. Though there is extensive research on the multimode-based stress state, most (but not all) researches have focused on the discrete stress state (e.g., stress state and non-stress state) and the model of global optimization. Taking a different approach, the presented paper focused on the transductive model based on the neighborhood knowledge, which could obtain better performance in the peripheral physiological dataset.

The major contributions can be concluded as follows:

1. We provided a transductive model, considering about the clustering methods based on neighborhood knowledge in high dimension.
2. A field study was presented in learning scenario, using non-linear features, based on non-invasive wearable device.

The former is about the transductive model, and the latter is about the learning scenario. Therefore, corresponding to the results section, two subsections are discussed in the remaining part of this section.

### 6.1. Discussion of Transductive Model

According to the results section, we can conclude three shortcomings from the DEAP and Stroop training caused by the individual differences, which are as follows: inter-class dispersed distribution, within-class irregular distribution, and sample imbalance in the DEAP. The first two relate to data distribution, while the last relates to label distribution. It exposes the common phenomena of obvious individualization and sample imbalance in the physiological signal databases. It leads to low prediction performance by the inductive reasoning methods. Therefore, subject-dependent training is generally the solution, whilst training based on all the subjects’ data cannot achieve a stable model. However, the proposed T-SVR using data from all the subjects for training had a better performance than the inductive method using subject-dependent training. It indicates the effectiveness of the proposed transductive method. Neighborhood knowledge helps the construction of a local regression, which is immune to outliers and irrelevant information, and it can supplement imbalance in samples from similar remaining subject data. In addition, the ST-SVR’s performance was slightly better than the T-SVR. This showed that even if there is no supplementation of the remaining subject data, the proposed method can ensure that the prediction error is small, and the influence of sample imbalance is smaller than the inductive reasoning method. Some research, e.g., Reference [3], can achieve the same goal but it is not suitable for real world application. This is because its central idea is to train and test after clustering, which leads to high errors if the test instance belongs to a category with few elements. Specific body conditions in different situations could also result in many feature categories with few elements.

Compared to the advanced study, ST-SVR achieved better performance in low stress recognition with smaller sliding window size. However, there are still some limits in the comparison with advanced methods, specifically:

(1) Few studies of stress recognition provided the description of the data distribution, which had a great influence on the recognition performance.
(2) The annotation method is different, including the self-report or automatic labelling.
(3) The difference in the underlying sensors causes the reliability of the received physiological signals to be unevaluable.

Therefore, we used the DEAP as a benchmark against our method, which provided small-grained labels and the same physiological signals (GSR and BVP), despite not being the exact same label. In the face of individualized differences, the state-of-the-art methods still used subject-dependent training. Although it seems that the subject-dependent performance was slightly better than the T-SVR, the development of its application in real-life scenarios is constrained. Its physiological signals are derived from short-term events, which cannot cover all possible physiological signals produced by individuals. Therefore, supplements from the rest of the samples may be necessary. From Section 5.1.4, we found that the performance of the DEAP was better than Stroop training, although its label distribution was imbalanced. Three reasons were noted as follows:

(1) The lower accuracy of the concurrent label than the self-assessment may be an important reason.
(2) Stroop training has a larger label granularity than the DEAP. Intuitively, the identified labels could also have larger intervals.
(3) The sliding window size was different. DEAP has a 1-minute signal length which may more fully characterize the psycho–physiological regular pattern.

Therefore, the establishment of a real-time physiological stress database still faces challenges.

In addition, the proposed method had a larger time cost in the ST-SVR and T-SVR than the other methods, except for the CNN. It is caused by the higher calculation cost. More specially, the T-SVR had a larger number of training samples than the other methods. However, in some real-time applications, the running time of the transductive model may be acceptable due to a minor delay (<0.1 s). It is worth noting that the differences in configuration of the operating platform and the number of examples could also produce the order of magnitude difference of time.

### 6.2. Discussion of Learning scenario 

Due to the CD values, it showed the reliability and realistic application possibilities of real-time stress recognition in this case. For real-time stress recognition, the student’s state could be recorded simultaneously with time. The viewing of offline data can help teachers or educators to identify the psycho–physiological patterns of students and to track how these patterns change over multiple learning sessions. Interaction can be through human intervention or fully automated. Therefore, this work helps to support teachers and students, especially the students from special groups using automatic e-learning in the future. But for applications with longer time, the objective unknown function with more variations than the number of training examples need to be considered. During the recognition processing or real use period, the label should be added when the observer finds a special state. The transductive model allows the observer to influence the subsequent recognition effects, which can be seen as a reinforcement of the learning process. This is one of our future research directions.

In addition, the difficult questions elicited the stress state much more easily, which confirmed the effectiveness of the experimental setup. And there was a puzzling phenomenon that the subjects obtained low ratio of correct answers to incorrect answers during the easy questions periods. The reason may be that subjects paid more attention and tried harder when they answered the difficult questions. The experimental results show that there could be a relationship between the scores and real-time stress, and we will specially study this problem in the future work.

## 7. Conclusions

Peripheral physiological signals-based stress recognition can promote the achievement of real-time stress recording using non-invasive wearable devices. The proposed transductive model was more effective for regression, and the performance was improved by clustering in high dimensionality. Thus, the presented ST-SVR and T-SVR had a better performance than the other methods. Experiments on the DEAP dataset and Stroop training confirmed the effectiveness of the proposed method. The real-time stress recognition in learning scenario was provided to explore the usability of the proposed framework for field studies. And the reliability of the real application was validated by retrospective assessment. Besides, there are still some limitations in this paper. For example, the subjects only had slight movement or were motionless, and the impact of vigorous movement to stress recognition was not considered. Relevant research will be carried out in the future work.

## Figures and Tables

**Table 1:** The features that can be extracted are listed.

Cha.	Feature	Description
BVP	Diastolic point (Low frequency)	The mean and SD of IBI
	Spectral features	The value of $c_{3}$ ^1^ in low frequency
	Statistical moments (Low frequency)	Mean and SD of signal
GSR	Peaks (High/Low frequency)	The number and amplitude of peaks
	Spectral features	The value of $c_{3}$ in low and high frequency
	Statistical moments (High/Low frequency)	Mean and SD of the first derivation, the second derivation, and negative slope [47]

^1^ Third order cumulant from the bi-spectrum analysis.

**Table 2:** Prediction results of the stress state using different methods. The best performances are highlighted using bold text.

		ε-SVR	Linear Regression	CNN	ST-SVR	T-SVR
Absolute Error	MAE	0.250	0.256	0.231	**0.193**	0.213
	SD	0.091	0.101	0.082	**0.061**	0.069
Time (ms)	Mean	3.69	**0.40**	322.90	63.34	121.67
	SD	0.20	**0.03**	43.16	11.29	38.92

**Table 3:** Prediction results of the valence using different methods. The best performances are highlighted using bold text.

		ε-SVR	Linear Regression	CNN	ST-SVR	T-SVR
Absolute Error	MAE	0.183	0.198	0.181	**0.156**	0.162
	SD	0.054	0.060	0.052	**0.043**	0.046
Time (ms)	Mean	**0.52**	1.01	201.55	28.98	41.53
	SD	**0.17**	0.67	50.14	9.34	13.67

**Table 4:** Quantitative prediction results of stress by the ST-SVR and T-SVR using the F-measure. The best performances are highlighted using bold text.

	QA-FSM	ST-SVR	T-SVR
Training Mode	Subject-dependent	Subject-dependent	Subject-independent
LS	0.943	**0.987**	0.929
MS	**0.970**	0.943	0.903
HS	**0.984**	0.975	0.969

**Table 5:** The Results of the Experiment in the Learning Scenario.

Standard of Question	Stress > 0.5	Stress ≤ 0.5	Correct	Incorrect	Answering Time(s)	CD	
	Number						
Difficult	388	217	577	28	Mean	17.26	85%
					SD	22.44	13.8%
Easy	101	194	200	95	Mean	15.80	84.95%
					SD	17.45	13.3%

**Figure A1:**
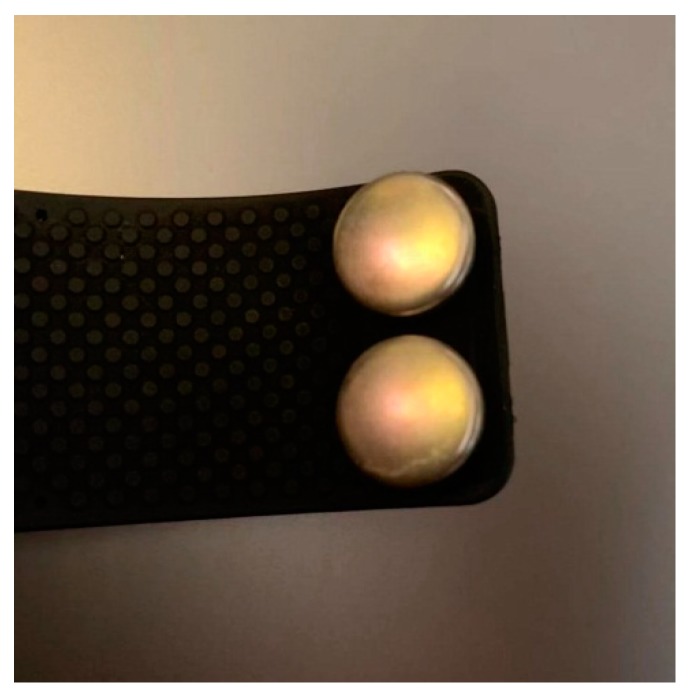
The Empatica electrodes.

**Figure A2:**
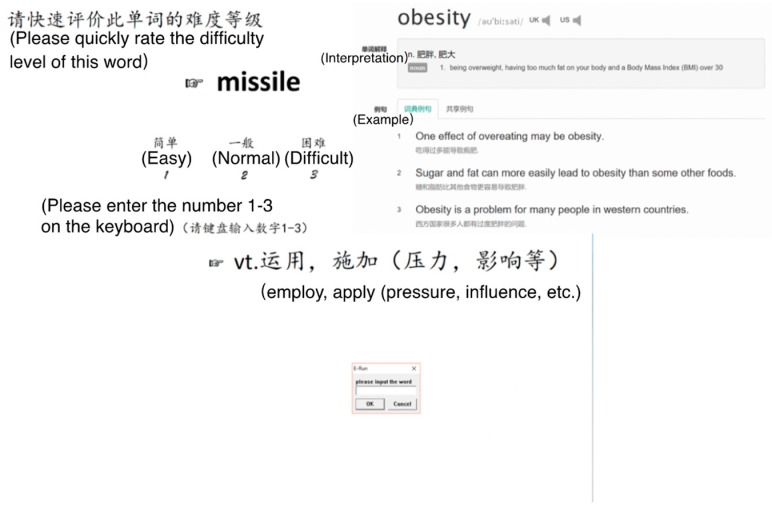
The interactive interface for learning English words.

## Appendix A

The Empatica wristband has 8 mm diameter silver coated with copper underlay on brass electrodes, as shown in Figure A1. The distance between the centers of the two probes is 1 cm.

## Appendix B

The interactive interface in learning scenario is shown in Figure A2, including three processes: showing the words without meaning, showing the words with meaning, and showing just the meaning of the words.
